# Distribution and prognostic relevance of tumor-infiltrating lymphocytes (TILs) and PD-1/PD-L1 immune checkpoints in human brain metastases

**DOI:** 10.18632/oncotarget.5696

**Published:** 2015-10-16

**Authors:** Patrick N. Harter, Simon Bernatz, Alexander Scholz, Pia S. Zeiner, Jenny Zinke, Makoto Kiyose, Stella Blasel, Rudi Beschorner, Christian Senft, Benjamin Bender, Michael W. Ronellenfitsch, Harriet Wikman, Markus Glatzel, Matthias Meinhardt, Tareq A. Juratli, Joachim P. Steinbach, Karl H. Plate, Jörg Wischhusen, Benjamin Weide, Michel Mittelbronn

**Affiliations:** ^1^ Edinger Institute, Institute of Neurology, University of Frankfurt am Main, Frankfurt am Main, Germany; ^2^ German Cancer Consortium (DKTK), Heidelberg, Germany; ^3^ German Cancer Research Center (DKFZ), Heidelberg, Germany; ^4^ Laboratory of Immunology and Vascular Biology, Stanford School of Medicine, Palo Alto, USA; ^5^ Department of Neurology, University of Frankfurt am Main, Frankfurt am Main, Germany; ^6^ Department of Neuroradiology, University of Frankfurt am Main, Frankfurt am Main, Germany; ^7^ Department of Neuropathology, University of Tuebingen, Tuebingen, Germany; ^8^ Department of Neurosurgery, University of Frankfurt am Main, Frankfurt am Main, Germany; ^9^ Department of Neuroradiology, University of Tuebingen, Tuebingen, Germany; ^10^ Senckenberg Institute of Neurooncology, University of Frankfurt am Main, Frankfurt am Main, Germany; ^11^ Department of Tumor biology, University Medical Center Hamburg-Eppendorf, Hamburg, Germany; ^12^ Institute of Neuropathology, University Medical Center Hamburg-Eppendorf, Hamburg, Germany; ^13^ Department of Pathology, University of Dresden, Dresden, Germany; ^14^ Department of Neurosurgery, Faculty of Medicine and University Hospital Carl Gustav Carus Technische Universität Dresden, Dresden, Germany; ^15^ Department of Gynecology, University of Wuerzburg, Wuerzburg, Germany; ^16^ Department of Dermatology, University of Tuebingen, Tuebingen, Germany; ^17^ Department of Immunology, University of Tuebingen, Tuebingen, Germany

**Keywords:** tumor-infiltrating lymphocytes, brain metastases, PD-1, PD-L1

## Abstract

The activation of immune cells by targeting checkpoint inhibitors showed promising results with increased patient survival in distinct primary cancers. Since only limited data exist for human brain metastases, we aimed at characterizing tumor infiltrating lymphocytes (TILs) and expression of immune checkpoints in the respective tumors.

Two brain metastases cohorts, a mixed entity cohort (*n* = 252) and a breast carcinoma validation cohort (*n* = 96) were analyzed for CD3+, CD8+, FOXP3+, PD-1+ lymphocytes and PD-L1+ tumor cells by immunohistochemistry. Analyses for association with clinico-epidemiological and neuroradiological parameters such as patient survival or tumor size were performed.

TILs infiltrated brain metastases in three different patterns (stromal, peritumoral, diffuse). While carcinomas often show a strong stromal infiltration, TILs in melanomas often diffusely infiltrate the tumors. Highest levels of CD3+ and CD8+ lymphocytes were seen in renal cell carcinomas (RCC) and strongest PD-1 levels on RCCs and melanomas. High amounts of TILs, high ratios of PD-1+/CD8+ cells and high levels of PD-L1 were negatively correlated with brain metastases size, indicating that in smaller brain metastases CD8+ immune response might get blocked. PD-L1 expression strongly correlated with TILs and FOXP3 expression. No significant association of patient survival with TILs was observed, while high levels of PD-L1 showed a strong trend towards better survival in melanoma brain metastases (Log-Rank *p* = 0.0537).

In summary, melanomas and RCCs seem to be the most immunogenic entities. Differences in immunotherapeutic response between tumor entities regarding brain metastases might be attributable to this finding and need further investigation in larger patient cohorts.

## INTRODUCTION

Interactions between immune and neoplastic cells play a major role during malignant progression nowadays being designated as the concept of cancer immunoediting [1]. Crucial phases in this concept of transformation from normal into neoplastic cells are sequentially subdivided into (I) an elimination phase, which allows the innate and adaptive immune system to destroy tumor cells before they become clinically conspicuous; (II) an equilibrium phase, which allows surviving (and presumably poorly immunogenic) cancer cells to rest in a dormant state; and (III) an escape phase that due to a constant selection pressure from immune cells enables tumor cells to mask thereby escaping the destruction by effector cells [1]. One mechanism of tumor cells to escape from killing by immune cells is to interfere with immune checkpoints, thereby disabling an adequate immune response [2]. Following this concept one promising target for an immune modulating therapy is the blocking of the Programmed cell death 1 (PD-1) / Programmed cell death ligand 1 (PD-L1) axis which is known to be crucial for immune escape mechanisms [3, 4]. PD-1 is expressed by activated lymphocytes and interactions of PD-1 with its ligand PD-L1, which is also expressed by neoplastic cells [5, 6] lead to a reduction of T-lymphocyte activity in normal tissue, during inflammation, in cancer and decreases autoimmunity [5, 7, 8]. These properties led to the development of therapies, blocking the PD-1/PD-L1 axis to intensify the anti-cancer immune response [9]. Two PD-1 antibodies were approved for advanced melanoma (pembrolizumab and nivolumab) or non-small cell lung carcinoma (NSCLC, nivolumab) during the last 12 months by the FDA and promising data on therapy response were also shown in small patient cohorts with renal cell carcinoma (RCC) [10–12]. A limiting factor for efficacy may be poor infiltration of lymphocytes into the tumor tissue [13–15].

Coming from the concept of cancer immunoediting, brain metastases range in the end stage of phase III - the escape phase of cancer cells. In the context of brain metastasis tumor cells even hide in an immune privileged microenvironment [16]. Brain metastases still constitute one of the most deleterious clinical conditions in tumor patients [17] and novel therapy strategies are urgently needed. For primary tumors or non-CNS-metastases, a meta-analysis has found that infiltration with CD8^+^ T cells correlated with improved survival in 58/60 studies [13]. Still, the association between density and localization of lymphocytic infiltrates and their prognostic value is an active field of research in many tumor entities [18–23]. However there is hardly any data on the prognostic values of TILs and the PD-1/PD-L1 axis in brain metastases.

The aim of our study was to provide a comprehensive overview about the distribution and phenotype of TILs in a large cohort of different brain metastases and compare it to clinical parameters such as patient overall survival and tumor size. Additionally, we provide expression data of central targets of immune checkpoint inhibitors such as the PD-1/PD-L1 system with regard to clinical parameters.

## RESULTS

### T-Lymphocytes infiltrate brain metastases in 3 distinct patterns

**Figure 1:**
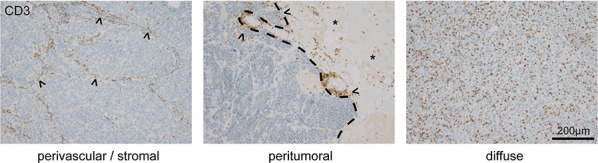
T-Lymphocyte infiltration patterns in human brain metastases Three distinct T-lymphocyte infiltration patterns are predominantly found in human brain metastases. The image on the left illustrates a perivascular infiltration of CD3-positive cells in the fibrovascular stroma (arrowheads) of a carcinoma. The middle image depicts a frequently observed peritumoral invasion pattern (arrowheads, dashed line showing the brain-tumor border of a carcinoma metastasis, asterisks pointing on reactive brain tissue). The image on the right illustrates the diffuse pattern of T-lymphocyte infiltration in a melanoma brain metastasis.

Infiltration of T-lymphocytes in brain metastases is mainly seen in 3 distinct patterns, although transitional patterns exist. Carcinomas which generate a prominent fibrovascular stroma such as NSCLC, breast carcinomas or colon carcinomas most often show a perivascular stromal infiltration of lymphocytes (Figure 1). Additionally, we frequently observed a peritumoral infiltration pattern of lymphocytes surrounding the tumor like a lymphocytic wall, which is mainly located in the surrounding brain tissue (Figure 1). As a third pattern most typically seen in melanomas, we found a homogenously diffuse lymphocytic infiltration of the tumor mass (Figure 1).

### Renal cell carcinomas and melanomas appear to be most immunogenic among brain metastases entities

**Figure 2:**
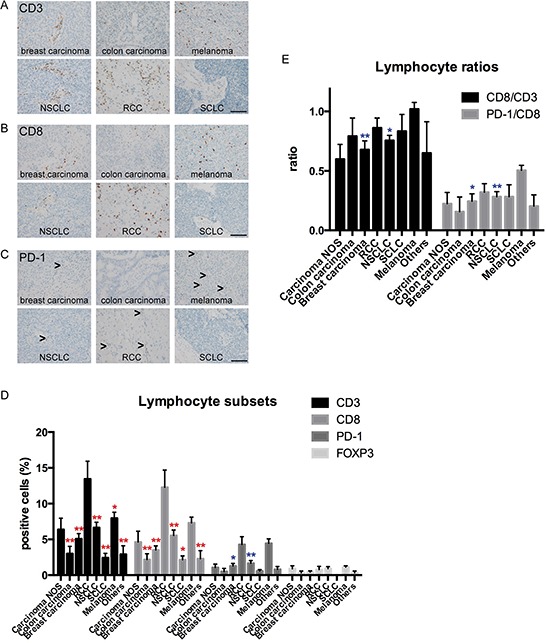
Comparison of TIL subtypes in different brain metastasis entities Representative stainings for tumor infiltrating lymphocyte subsets in human brain metastases are depicted for CD3 **A.** CD8 **B.** and PD-1 **C.** Arrowheads indicate infiltrating lymphocytes (scale bars 100 μm). **D.** showing quantification of CD3-, CD8-, PD-1 and FOXP3-positive lymphocytes in different entities. The respective percentages of CD3-, CD8-, PD-1- and FOXP3-positive lymphocytes are indicated. Red asterisks show significant results compared to RCCs (*p < 0.05; **p < 0.01). Blue asterisks show significant results compared to melanomas. All other comparisons did not reveal significant differences. In **E.** the ratios between CD8/CD3 and PD-1/CD8 cells are depicted. Blue asterisks indicate significant differences compared to melanomas (*p < 0.05; **p < 0.01).

The amount of tumor infiltrating lymphocytes was highly variable with pronounced infiltration in RCCs and melanomas. First, we investigated the amount of CD3-positive lymphocytes as a reference for pan-T-cell-infiltration. Here we found statistical differences between the different entities, with RCCs showing a strongly pronounced T-lymphocytic immune response (CD3+ lymphocytes; mean: 13.4%) (Figure 2D). Brain metastases of SCLC and colon carcinomas showed only very low amounts of CD3-positive lymphocytes in the tumor tissue (SCLC: CD3+ lymphocytes; mean: 2.4%; colon carcinoma: CD3+ lymphocytes; mean: 3.0%; Figure 2D). To quantify potential effector cells, we additionally analyzed the amount of CD8-positive lymphocytes in the different brain metastases entities. The amount of CD8-positive lymphocytes was significantly higher in brain metastases of RCCs (mean: 12.8%) as compared to almost all other entities except melanomas and undifferentiated carcinomas, which were not otherwise specified (NOS) (Figure 2D). As current therapeutic approaches target the checkpoint PD-1/PD-L1 axis we additionally analyzed PD-1-positive lymphocytes in tumor specimens. As CD8-positive TILs are known to express high levels of PD-1 and thus may be functionally impaired in the presence of PD-L1 expression [24, 25] we generated the ratio between PD-1 and CD8 in the different brain metastases entities (Figure 2E). We found the highest amount of PD-1-positive lymphocytes in melanomas (mean: 4.4%) and RCCs (mean 4.3%). Melanoma brain metastases even showed a statistically significant higher amount of PD-1-positive lymphocytes as compared to brain metastases of NSCLC and breast carcinomas. The ratio between PD-1-positive lymphocytes and CD8-positive effector cells was highest in melanomas (mean ratio: 0.51). In spite of the relatively low number of samples, this difference even turned out to be significant when compared to NSCLC and breast carcinomas (Figure 2E). The amount of FOXP3-positive cells was generally low among all entities (Figure 2D).

### Number of TILs and PD-1/CD8 ratio negatively correlates with the size of brain metastases

**Figure 3:**
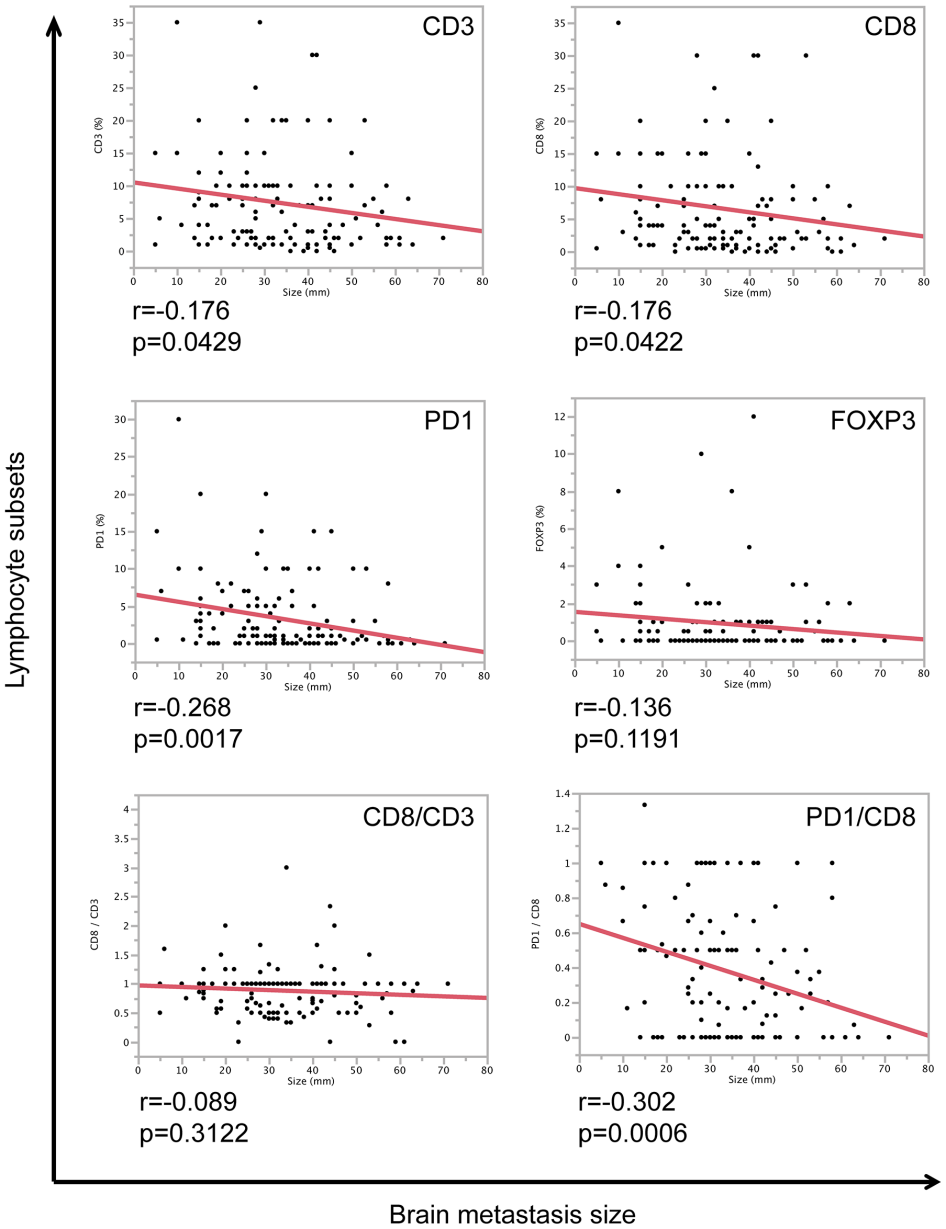
Correlation of TILs and brain metastasis size Correlation analyses of brain metastasis size (mm) and lymphocyte subsets (CD3 (%), CD8 (%), PD-1 (%), FOXP3 (%) as well as CD8/CD3 and PD-1/CD8 ratios). *r*-values and *p*-values of correlation analyses are depicted below each graph.

We next asked the question whether the size of brain metastases is associated with the amount of TILs. We were able to measure the size of 148 brain metastases patients using T1-weighted MRI sequences with contrast agent. Interestingly, both CD3- and CD8-positive lymphocytes showed a mild significant negative correlation with brain metastases size (Figure 3). Surprisingly, PD-1-positive lymphocytes as well as the ratio between PD-1- and CD8-positive lymphocytes were strongly negatively correlated with brain metastases size (Figure 3), meaning smaller brain metastases showed an increased ratio of PD-1/CD8, which might explain an early immune escape mechanism in the brain.

Since melanomas presented with highest levels of PD-1 as well as the highest PD-1/CD8 ratio among all tested entities, we additionally compared TILs and brain metastases size in melanoma patients. Although we did not find a significant correlation between tumor size and TILs (CD3: *r* = 0.031, *p* = 0.7968; CD8: *r* = 0.0223, *p* = 0.8648) in melanomas, still the PD-1/CD8 ratio was strongly negatively correlated with brain metastases size (*r* = 0.3331, *p* = 0.0093) (data not shown).

### TILs are not associated with patient survival in brain metastases

**Figure 4:**
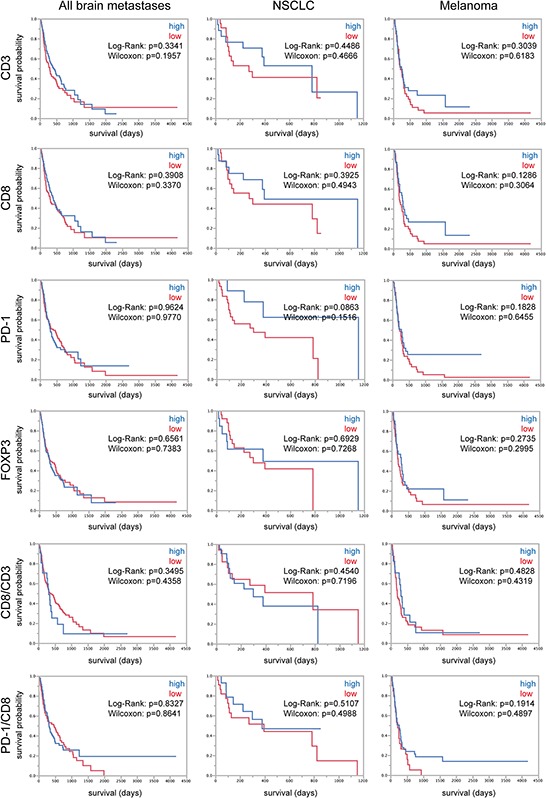
TILs and survival of brain metastasis patients Kaplan-Meier curves depicting patient survival in the whole cohort of all brain metastases patients and the subcohorts of NSCLC and melanoma patients. High and low expressers were stratified after median split.

Brain metastases are associated with a very bad prognosis, nevertheless some patients show a better clinical outcome as others. Therefore molecular and/or cellular predictors of patient survival are strongly needed. For that reason we analyzed our patient cohorts regarding the association of TILs and patient overall survival after surgical excision of the tumor. We stratified for low and high expressers of each single factor and the associated ratios. First, we analyzed the whole mixed cohort of all patients without stratification for tumor subtype (follow-up data of *n* = 204 patients), then, as we were interested in analyzing large homogenous cohorts we picked out the cohorts, of which we were able to record most survival data, i.e. NSCLC (follow-up data of *n* = 44 patients) and melanoma patients (follow-up data of *n* = 91 patients) (Figure 4). In the whole cohort of patients as well as in the two subcohorts of NSCLC and melanoma we did not find an association between TILs and patient overall survival (Figure 4). As TILs were reported to play a prognostic role in breast carcinomas we additionally analyzed a second cohort exclusively containing breast carcinoma brain metastases of *n* = 96 patients (follow-up data of *n* = 42 patients) (Supplementary Figure S1). Although we found a prognostic role for CD8-positive cells in this cohort (Supplementary Figure S1), the statistical analysis was not significant when we added breast carcinoma follow-up data of the first cohort to this breast carcinoma cohort (Log-Rank *p* = 0.1538, Wilcoxon *p* = 0.2665) (data not shown).

### PD-L1 expression strongly correlates with FOXP3-positive lymphocytes in brain metastases

**Figure 5:**
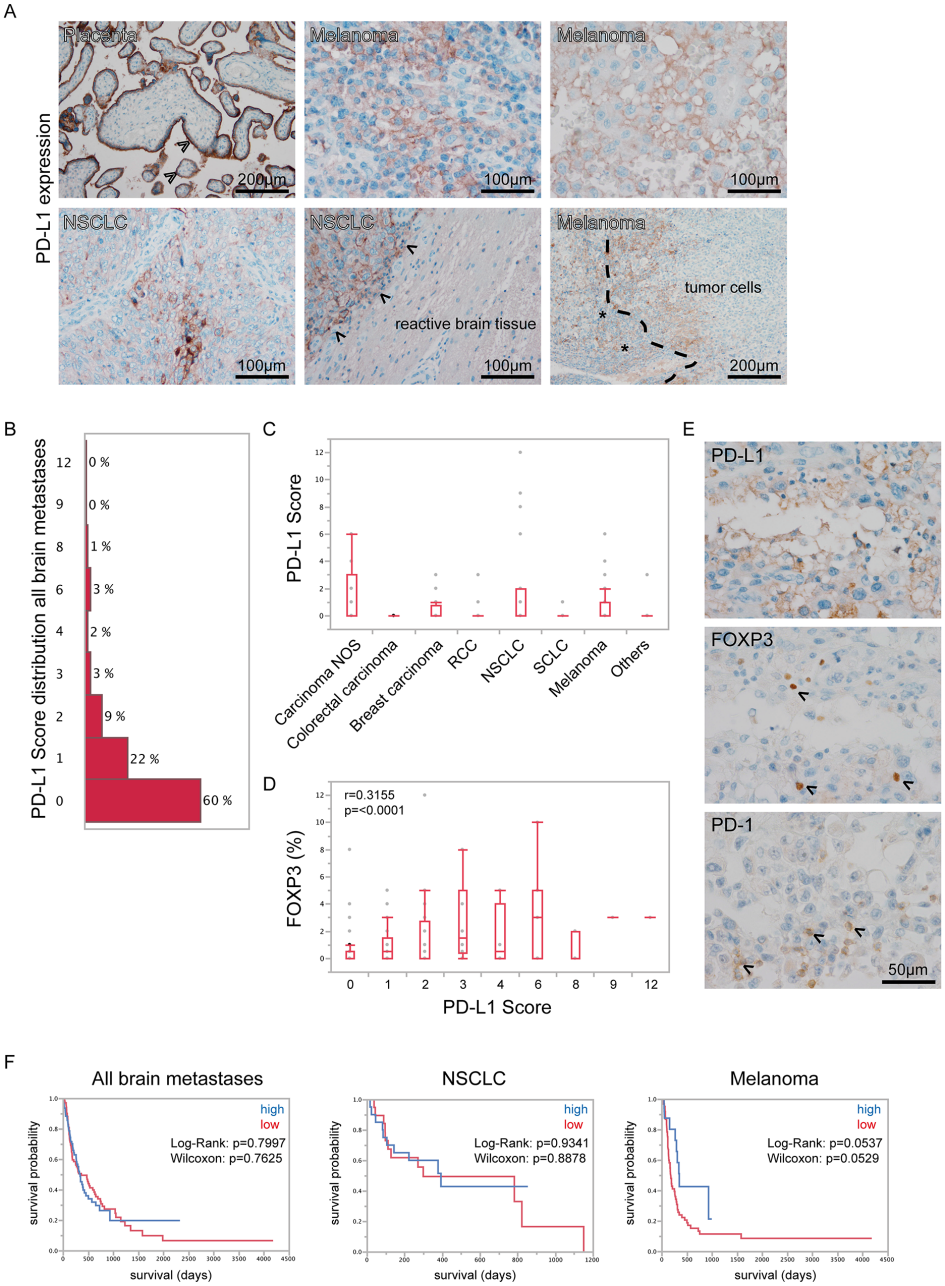
PD-L1 expression in human brain metastases **A.** Immunohistochemical stainings for PD-L1 revealed strongest expression on placental syncytiothrophoblasts (arrowheads upper left), while throughout the different specimens expression appeared heterogeneous. On tumor cells, we observed a membraneous staining while adjacent reactive brain tissue was PD-L1-negative (arrowheads lower middle indicating tumor-brain border). In some specimens we found a loco-regional overlap of TILs (asterisks lower right) and PD-L1 expression in tumor cells (dashed line lower right indicating tumor-lymphocyte border). In **B.** the distribution of the PD-L1 score among all brain metastases is displayed (median value = 0). In **C.** box-plots of PD-L1 score throughout all entities are shown. No statistically significant differences were observed. In **D.** box-plots of PD-L1 scores with corresponding FOXP3 expression in brain metastases are depicted. Results of Spearmans Rho correlation analysis are shown in the chart. **E.** PD-L1, FOXP3 and PD-1 expression are shown in the same area of a melanoma brain metastasis. **F.** Kaplan-Meier survival curves were generated after stratification into high and low PD-L1 expressers using a median split.

To complete the analysis of the PD-1/PD-L1 system, we additionally analyzed the expression of PD-L1 in our brain metastasis specimens. We detected strong PD-L1 expression in placental syncytiotrophoblasts serving as a positive control (Figure 5A). In general the expression of PD-L1 was rather low and, in fact, undetectable in >50% of the investigated brain metastasis specimens. We found highest absolute levels of PD-L1 in NSCLC, whereas melanomas showed the highest median PD-L1 expression (score 1, Figure 5B and 5C). Interestingly, PD-L1 showed a significantly strong correlation with TILs (Table 1).

**Table 1:** Correlation analysis of PD-L1 expression and lymphocyte subsets

	CD3 all	CD3 NSCLC	CD3 Melanoma	CD8 all	CD8 NSCLC	CD8 Melanoma
PD-L1 all	ρ = 0.4447 *p* < 0.0001			ρ = 0.4471 *p* < 0.0001		
PD-L1 NSCLC		ρ = 0.5615 *p* < 0.0001			ρ = 0.5414 *p* < 0.0001	
PD-L1 Melanoma			ρ = 0.4155 *p* < 0.0001			ρ = 0.4003 *p* = 0.0002

Results of Spearman's Rho correlation analysis are depicted in the table.

As PD-L1 expression is known to increase the amount of FOXP3-positive lymphocytes [26] we analyzed whether there is a correlation between PD-L1 expression by tumor cells and the amount of FOXP3-positive lymphocytes. We found a significantly positive correlation of PD-L1 expression and the frequency of FOXP3-positive lymphocytes, leading to the assumption that the functional system of PD-1/PD-L1 interactions might be active in brain metastases (Figure 5D).

We neither found significant associations between high or low PD-L1 expressers and patient overall survival in the whole brain metastasis cohort nor in the subcohorts of NSCLC and melanomas (Figure 5E) as well as in the second exclusive breast carcinoma brain metastases cohort (Supplementary Figure S1). Nevertheless melanoma patients showed a strong trend towards a rather better survival when expressing higher levels of PD-L1.

**Figure 6:**
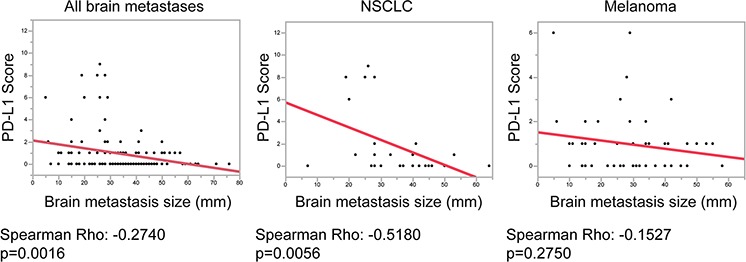
Correlation analyses of PD-L1 expression and brain metastasis size Graphics depicting results of linear fit between PD-L1 score and brain metastases size in mm. All brain metastases of the whole cohort, as well as the subcohorts NSCLC and melanomas are shown. *P*-values and Spearman's Rho correlation coefficients are shown below the graphs.

Although we did not find significant differences in patient survival with regard to PD-L1 expression, we were interested whether we could observe a similar negative correlation of PD-L1 with brain metastasis size as we did for the PD-1/CD8 ratios. Interestingly, in the whole brain metastasis cohort we detected a strong negative correlation of PD-L1 with brain metastasis size (*p* = 0.0016; ρ = −0.2740) (Figure 6) with highest PD-L1 levels in brain metastases smaller than 30 mm in diameter. Similar results were detected in NSCLC patients (*p* = 0.0056; ρ = −0.5180) (Figure 6). Nevertheless, in the subcohort of melanoma brain metastases we did not observe a significant correlation of PD-L1 with brain metastases size (*p* = 0.2750; ρ = −0.1527).

## DISCUSSION

Brain metastasis constitutes a devastating clinical situation, being associated with severe patient disability leading to a poor survival prognosis. Standard treatment strategies are difficult to establish, as each patient presents with an individual treatment history, systemic tumor status and tumor subtype. There are only few possibilities to estimate patient prognosis, mainly taking into account clinical parameters such as age or Karnofsky performance status (KPS) [17, 27] (see also Supplementary Figure S2). Until today only few molecular biomarkers predicting patient survival have been described [28, 29].

Since several decades the lymphocytic immune response, namely TILs are considered to be associated with patient prognosis. Recently, several studies independently described a significant association of TILs with better patient survival in primary NSCLC, melanoma and breast cancer [20, 21, 23, 30]. Some authors recommended to include the grade of TILs in the pathological reports as the amount of TILs was even predictive for therapy response [21]. So far there is only little data on the distribution and prognostic relevance of TILs in human brain metastases. Recently, Berghoff and colleagues analyzed 43 melanoma brain metastases for TILs and PD-L1 expression with regard to patient survival. In their study the authors did not find an association between TILs and patient overall survival after diagnosis of brain metastases [31].

In the present study, we analyzed a large cohort of 252 brain metastases patients with regard to TILs and subtypes, as well as to PD-1 and PD-L1 expression. Neither in the whole cohort of brain metastases nor in the larger subcohorts of melanomas and NSCLC did we find a significant association of TILs and patient survival. As TILs have been reported to be of prognostic relevance also in patients with breast carcinomas, we additionally analyzed a breast carcinoma brain metastasis cohort. Interestingly, in this small cohort with survival data of *n* = 42 patients we found a prognostic impact of CD8-positive lymphocytes with better patient prognosis in case of higher amounts of CD8-positive cells. Nevertheless, this finding should not be overrated, as this statistically significant result was not robust when adding the CD8 results and survival data of breast carcinomas of our first cohort (Log-Rank *p* = 0.1538, Wilcoxon *p* = 0.2665). One technical explanation for these results might be the differences of TMA core size we used for these two cohorts. While the TMAs of the first cohort (*n* = 252 patients) were generated with large 2 mm diameter punches, covering a tumor area of 3.14 mm^2^, the second cohort consisted of small 0.6 mm punches covering a tumor area of 0.28 mm^2^. Because of these technical differences, we decided not to combine these two cohorts and kept them separately (for details see Table 2 and materials and methods section).

**Table 2:** Clinical data of the mixed brain metastasis cohort

Primary tumour	Sex m/f	Patient age (years)	KPS	No. of BM	Size of BM (mm)	Follow-up (days)
**Carcinoma** **NOS** **(*n* = 11)**	2/9	48–73 median 63	40–90 median 70 *n* = 10/11	1–13 median 1 *n* = 10/11	15–73 median 41 *n* = 10/11	27–428 median 155 *n* = 7/11
**Colorectal** **carcinoma** **(*n*= 11)**	7/4	56–73 median 66	30–100 median 80 *n* = 8/11	1–16 median 18/11	23–48 median 367/11	35–1346 median 352 *n* = 8/11
**Breast** **carcinoma** **(*n* = 33)**	–/33	32–76 median 58	30–100 median 80 *n* = 20/33	1–20 median 2 *n* = 20/33	23–76 median 36 *n* = 18/33	6–1331 median 492 *n* = 24/33
**RCC** **(*n* = 18)**	17/1	45–76 median 65	30–100 median 90 *n* = 8/18	1–3 median 1 *n* = 9/18	20–45 median 30 *n* = 7/18	18–1985 median 536 *n* = 15/18
**NSCLC** **(*n* = 62)**	36/26	13–80 median 61	20–100 median 80 *n* = 34/62	1–10 median 1 *n* = 34/62	7–64 median 36 *n* = 30/62	13–1276 median 260 *n* = 44/62
**SCLC** **(*n* = 9)**	4/5	43–72 median 57	30–90 median 70 *n* = 8/9	1–8 median 1 *n* = 8/9	32–71 median 548/9	14–653 median 364 *n* = 7/9
**Melanoma** **(*n* = 98)**	56/42	20–83 median 60	30–100 median 8067/98	1–20 median 1 *n* = 80/98	5–61 median 28 *n* = 63/98	16–4166 median 183 *n* = 91/98
**Others** **(*n* = 10)**	6/4	33–73 median 54	30–100 median 80 *n* = 7/10	1–4 median 2 *n* = 7/10	18–44 median 32 *n* = 5/10	47–744 median 232 *n* = 8/10
**TOTAL** **(*n* = 252)**	128/124	13–83 median 61	20–100 median 80 *n* = 162/252	1–20 median 1 *n* = 176/252	5–76 median 33 *n* = 148/252	6–4166 median 245 *n* = 204/252

Abbreviations: m: male; f: female; KPS: Karnofsky Performance Status; BM: brain metastases.

Along this line, a criticism that should be discussed at this point is the usage of TMAs for the evaluation of TILs. For validation of our results we applied a double variability control strategy using on the one hand repetitive punches of vital tumor parts from different sites of the paraffin block, on the other hand we generated a validation TMA with several punches from the same tumor and compared these results with the analyses of TILs in the whole mount sections. We found highly significant, strongly positive correlations between TILs in the different variable punches as well as in comparison with the whole mount sections (for details see Supplementary Figure S2C and S2D). Thus, the TMAs we generated are a useful tool for TILs analyses.

Throughout all different entities we found the highest amount of TILs in RCCs and highest ratios of PD-1/CD8-positive lymphocytes in melanomas, indicating that RCCs and melanomas might be most immunogenic of the common brain metastases entities. As RCC brain metastases constitute the group with the highest vascular density [32] this statement should be handled with care, as the lymphocytic infiltrate might be associated with increased perivascular lymphocyte deposits. Interestingly, melanoma brain metastases are the brain metastases entity with lowest vascular density [32] but still the amount of TILs is quite high as compared to other groups with low angiogenic potential such as brain metastases of SCLC. Thus, melanomas might be the most immunogenic of the common brain metastasis entities. A major criticism sometimes arising when analyzing TILs in brain tumors is to which extent patients' pre-surgical corticosteroid treatment may affect the amount of TILs. Interestingly, corticosteroids neither affect the amount of TILs nor PD-L1 expression in melanoma brain metastases [31]. The hypothesis that melanoma brain metastases might be most immunogenic was reflected by a prominent diffuse lymphocytic infiltration in our cohort. To get an idea whether TILs can contain the size of brain metastases, we analyzed our large cohort of brain metastasis in detail and found that the amount of TILs was generally associated with metastasis size, i.e. smaller metastases showed an increased infiltration of TILs. Interestingly, the ratio between PD-1- and CD8-positive lymphocytes showed a significant negative correlation with metastases size, meaning that highest ratios were present in smaller metastases. Along this line, also PD-L1 expression was highest in smaller brain metastases in the whole cohort and in the subcohort of NSCLC, showing a negative correlation with brain metastasis size. This might give new insights in the dynamics of lymphocyte infiltration in brain metastases, and might indicate that in smaller metastases the lymphocytic immune response is activated but might be functionally impaired. Alternatively, T cells might have contained the tumor size for a while before becoming exhausted (which is indicated by high levels of PD-1). In the same sense - although not reaching the level of statistical significance - FOXP3-positive lymphocytes were increased in smaller brain metastases. Supporting data for these findings derives from a clinical study in which patients with advanced melanoma and brain metastases were treated with the checkpoint-inhibitor Ipilimumab (anti-CTLA-4 antibody) showing antitumor activity especially in patients with smaller asymptomatic brain metastases [33].

As the activation status of lymphocytes is strongly dependent on PD-L1 we additionally analyzed the tumor specimens for PD-L1 expression. PD-L1 is induced by multiple inflammatory stimuli such as IFN-γ or TNF-α [34] and is transcriptionally regulated by PI3K-pathway, NF-κB, NPM (nucleophosmin) / ALK (anaplastic lymphoma kinase) and STAT3 [4, 35, 36]. In line with the generally low level of inflammatory stimuli in the brain, the expression of PD-L1 in the tumor specimens was rather low, with more than 80% of the investigated tumors presenting with less than 10% positive tumor cells. Interestingly, we focally found a loco-regional overlap between TILs and PD-L1 expression. Additionally we observed a strong correlation between PD-L1 expression and TILs. This local association might be explained by an induction of PD-L1 in tumor cells by infiltrating lymphocytes which are known to be a source of interferon-gamma [34]. Interestingly, this loco-regional association of TILs and PD-L1 expression has already been described in non-brain metastatic melanomas which most likely represents a kind of “adaptive immune resistance” mechanism [37]. Interestingly, Taube and colleagues reported prolonged patient survival in metastatic melanoma when tumor cells expressed high levels of PD-L1 [37]. In the present study we were able to demonstrate that PD-L1 expression was heterogeneous among the different tumor entities with absolute highest expression levels in NSCLC and highest median expression in melanomas. When analyzing patient survival with regard to PD-L1 expression, we did not find a significant survival benefit neither in the total cohort of brain metastases nor in the subcohorts of NSCLC and melanoma. Nevertheless, melanoma patients showed a strong trend reaching almost statistical significance towards a better survival when expressing high PD-L1 levels, which is in line with the observations in non-brain melanoma metastases [37].

In spite of some cohorts showing a trend towards better survival in case of higher amounts of TILs or high PD-L1 expression, our data do not show clear evidence for a prognostic role of TILs in human brain metastases. This supports the hypothesis that anti-tumoral immune responses may be more effective at preventing metastasis than at eradicating already established metastases [38, 39]. Moreover, comparing tumor: immune cell interactions in brain metastases allow for unprecedented comparisons regarding the intrinsic immunogenicity of the various tumor types without having to adjust for contributions from the respective microenvironments. Our study hence complements very recent analyses which have assessed the level of immunogenicity for various tumor entities based on the average number of genetic mutations, on the stochastic or disproportionate ratio between immunogenic and non-immunogenic mutations (classified by their ability to be presented on HLA) and on Granzyme A and Perforin mRNA expression in the primary tumor tissue [40, 41]. These approaches revealed breast cancer to be poorly immunogenic due to a low number of mutations [40]. NSCLC harbors a roughly 5-fold larger number of non-synonymous mutations which are essential determinants for the response to immunotherapy with anti PD-1 antibodies [42]. Cytolytic activity in lung cancer tissue was, consequently, much higher but still below the level observed in the healthy lung. This may indicate that the tumor cells actively suppress immune responses. The most immunogenic tumors were, in contrast, renal cell carcinomas. Significant cytolytic activity was also observed for T cells in melanomas, even though the tumors themselves did not seem to have undergone much immunoediting. Strikingly, our analysis of brain metastases revealed a similar pattern of immunogenicity with renal cell carcinoma being most immunogenic, followed by melanoma whereas brain metastases from breast cancer and NSCLC were hardly infiltrated by immune cells. This indicates that the previously described differences between these tumor entities were, in fact, due to intrinsic properties of the tumor cells and not to the level of immunoprotection conferred by the tumor microenvironment. Conversely, the similarity between these patterns suggest that the parameters which are critical for anti-tumoral immune responses in primary tumors are also relevant for brain metastases. Accordingly, the idea that therapeutic manipulation of immune checkpoints may not only increase immune responses in the periphery, but also prevent macrometastasis formation in the human brain, appears quite plausible. This, however, remains to be confirmed by future clinical studies.

## MATERIALS AND METHODS

### Tissue specimen and tissue processing

We analyzed formalin-fixed and paraffin-embedded (FFPE) tissue from archived brain metastases, which we collected and processed as tissue micro arrays (TMAs). The paraffin blocks for TMA generation were collected between the years 1999 and 2014. All blocks were stored at room temperature. Antigenicity of tissue used for the TMAs was tested using standard diagnostic markers for proliferation and intermediate filaments such as Ki67 and different cytokeratins. All specimens were obtained either from the UCT tumor bank (Goethe-University, Frankfurt am Main, Germany, member of the German Cancer Consortium (DKTK), Heidelberg, Germany and German Cancer Research Center (DKFZ), Heidelberg, Germany) or from the local biobank “Blut-und Gewebebank zur Erforschung des malignen Melanoms” (Department of Dermatology, University Hospital Tuebingen, Tuebingen, Germany). Approval for this study was conferred by two independent ethical committees (Ethics committee UCT Frankfurt / Goethe University Frankfurt am Main, Germany: project numbers: GS 4/09; SNO_01-12; Ethics committee University of Tübingen project number: 183/2015BO2). We investigated one large, mixed cohort of brain metastases (clinical data on this cohort is depicted in Table 2) and a second validation cohort exclusively consisting of breast cancer brain metastases. From this cohort we were able to analyze 96 patients. Follow-up clinical survival data was recorded from 42 of the 96 analyzed patients. Both cohorts were analyzed separately. Results of survival analyses of the validation cohort are depicted in Supplementary Figure S1.

In the large mixed cohort, we investigated 252 patients with brain metastases including: melanoma (*n* = 98), NSCLC (*n* = 62), breast carcinoma (*n* = 33), RCC (*n* = 18), SCLC (*n* = 9), colon carcinoma (*n* = 11), carcinomas which were not otherwise specified (carcinoma NOS *n* = 11) and specimens of rare tumors summarized as others (*n* = 10). We collected follow-up survival data of 204 patients (survival time after tumor resection) of the mixed cohort. For validation of known survival factors in our brain metastases cohort we obtained KPS in 162 of 252 patients. Additionally, we analyzed the “Graded Prognostic Assessment”-Score (GPA-Score) in our melanoma cohort. Results are shown in Supplementary Figure S2A and S2B.

### Neuroradiology

T1-weighted MRI-sequences with contrast agent before neurosurgical intervention were analyzed by experienced neuroradiologists (SB and BB) as well as trained investigators (MK and RB). The number of brain metastases was analyzed in 176 patients. Brain metastases size was measured in 148 patients. The largest diameter of the contrast enhancing mainly round structures corresponded to the measured brain metastasis size (for details see also Table 2).

### Immunohistochemistry

Immunohistochemistry for all antibodies was performed using 3 μm thick slides and standard protocols on the automated IHC staining system Discovery XT (Roche/Ventana, Tucson, Arizona, USA). The following antibodies were used: CD3 (A0452, dilution 1:500, DAKO, Glostrup, Denmark), CD8 (clone C8/144B, dilution 1:100, DAKO, Glostrup, Denmark), PD-1 (clone NAT105; dilution 1:50; Abcam, Cambridge, United Kingdom), PD-L1 (E1L3N; dilution 1:200; Cell Signaling, Boston, U.S.A.), FOXP3 (clone 236A/E7; dilution 1:100; eBioscience, San Diego, U.S.A.) Slides were counterstained with hematoxylin and mounted.

### Statistical analyses

All samples were scored according to the frequency of positive cells related to all cells (as percentage) on the stained TMA core. For PD-L1 expression we used a score as previously described in detail [28, 43]: frequency 0–1% score 0; 1–10% score 1; 10–25% score 2; 25–50% score 3; >50% score 4; additionally we multiplied the frequency score with the intensity of staining (1 weak staining, 2 moderate staining, 3 strong staining), finally resulting in the ordinal scaled PD-L1 score (0, 1, 2, 3, 4, 6, 8, 9, 12). Ordinal scaled variables were compared with non-parametric Wilcoxon/Kruskal-Wallis-Test and Dunn`s method to correct for multiple testing. For continuous variables, means were compared between different brain metastases entities using ANOVA, followed by Tukey-Kramer HSD post-hoc Test. For correlation analyses of brain metastases size and marker expression we performed a linear fit followed by ANOVA, in case of ordinal scaled variables we used Spearman's rho correlation analysis. Patient survival was assessed by Kaplan-Meier analyses after stratification into high and low expressers of each tested variable such as CD3, CD8, FOXP3, PD-1 and PD-L1. For statistical analyses of patient survival we used Log-Rank and Wilcoxon testings. A significance level of *p* < 0.05 was set for all statistical analyses.

All statistical analyses were performed using JMP8 and JMP11 (SAS, Cary, U.S.A.), additional graphics were created with Prism 6 (GraphPad Software, La Jolla, U.S.A.).

## SUPPLEMENTARY FIGURES


