# Supplementary material for: Distribution and prognostic relevance of tumor-infiltrating lymphocytes (TILs) and PD-1/PD-L1 immune checkpoints in human brain metastases
Source: Oncotarget. 2015 Oct 16;6(38):40836–49. doi: 10.18632/oncotarget.5696 (PMC4747372; doi:10.18632/oncotarget.5696)
Supplement: Supplementary file 1 [file oncotarget-06-40836-s001.pdf]

SUPPLEMENTARY FIGURES

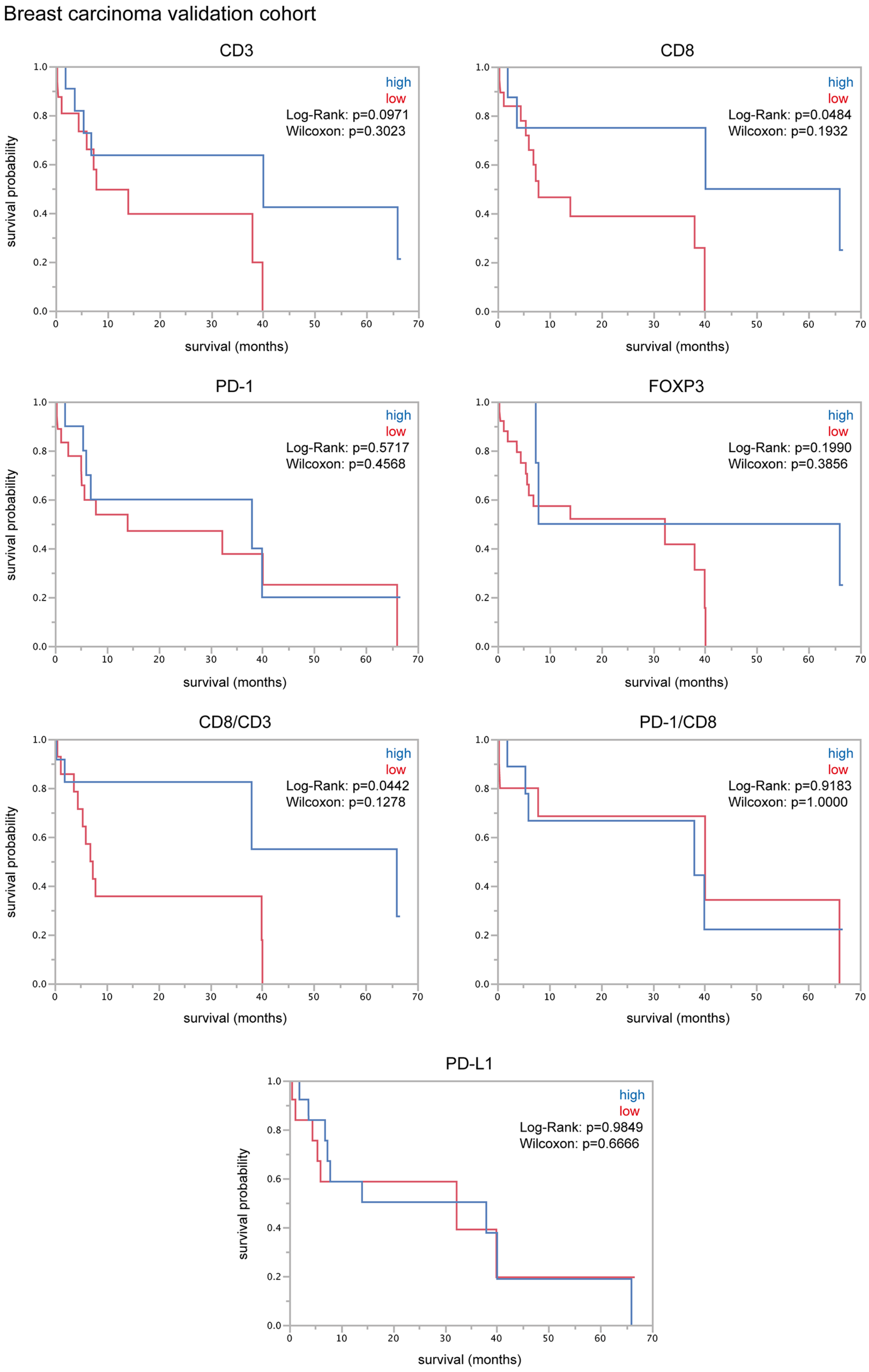

Supplementary Figure S1: Kaplan-Meier survival analysis of the exclusive breast carcinoma brain metastasis validation cohort.

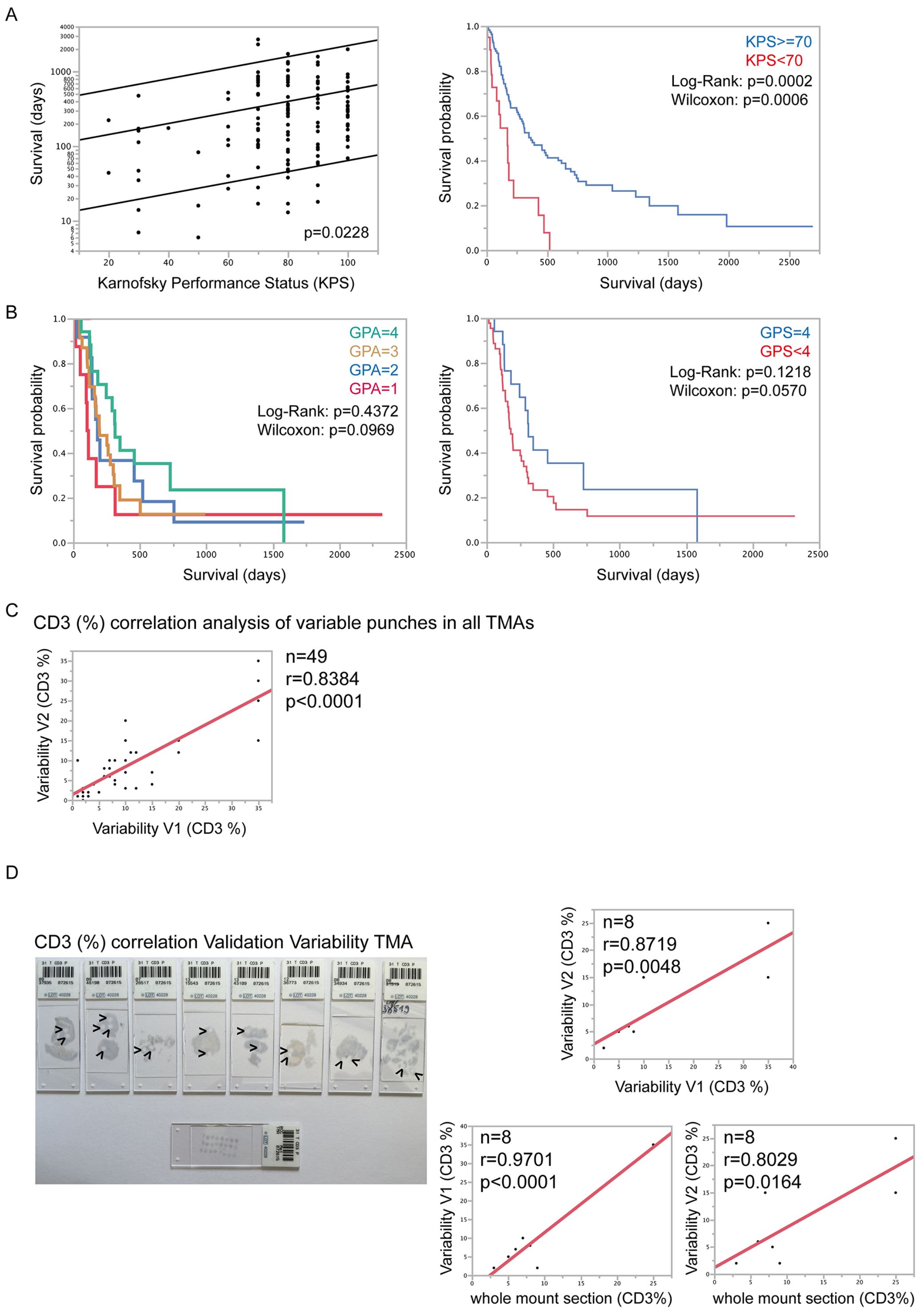

Supplementary Figure S2: Survival data and tumor specimen variability analyses of the brain metastasis cohort. **A.** Results of parametric survival analyses (left graph) and Kaplan-Meier survival analysis stratified with regard to KPS (right graph). **B.** Kaplan-Meier survival analyses of melanoma brain metastases with regard to GPA-Scores (including number of brain metastases and KPS). **C.** Correlation analysis of TILs (CD3-positive lymphocytes) in variable punches (V1 and V2) of the same tumor of the whole cohort. **D.** Overview of the generation of a validation cohort of RCC brain metastases. Arrowheads point on punched cores of the tissue specimens in the whole mount section. Right graphs illustrating correlation analyses of TILs (CD3-positive lymphocytes) between variable punches (V1 versus V2, upper graph) and correlation analyses between the variable punches (V1 and V2) and the corresponding whole mount sections (lower graphs).
